# Thermal Expansion of 3C-SiC Obtained from In-Situ X-ray Diffraction at High Temperature and First-Principal Calculations

**DOI:** 10.3390/ma15186229

**Published:** 2022-09-08

**Authors:** N. M. Sultan, Thar M. Badri Albarody, Husam Kareem Mohsin Al-Jothery, Monis Abdulmanan Abdullah, Haetham G. Mohammed, Kingsley Onyebuchi Obodo

**Affiliations:** 1 Department of Mechanical Engineering, Universiti Teknologi PETRONAS (UTP), Bandar Seri Iskandar 32610, Malaysia; 2 Mechanical Engineering Department, University of Al-Qadisiyah, Al-Diwaniyah 58001, Qadisiyah, Iraq; 3 HySA Infrastructure Centre of Competence, Faculty of Engineering, North-West University (NWU), Potchefstroom 2531, Northwest Province, South Africa

**Keywords:** thermal expansion isotropy, X-ray diffraction, DFT calculation, CASTEP, SiC

## Abstract

In situ X-ray crystallography powder diffraction studies on beta silicon carbide (3C-SiC) in the temperature range 25–800 °C at the maximum peak (111) are reported. At 25 °C, it was found that the lattice parameter is 4.596 Å, and coefficient thermal expansion (CTE) is 2.4 ×$10^{-6}$/°C. The coefficient of thermal expansion along a-direction was established to follow a second order polynomial relationship with temperature $(\alpha_{11}=-1.423\times 10^{-12}T^{2}+4.973\times 10^{-9}T+2.269\times 10^{-6}$). CASTEP codes were utilized to calculate the phonon frequency of 3C-SiC at various pressures using density function theory. Using the Gruneisen formalism, the computational coefficient of thermal expansion was found to be 2.2 ×$10^{-6}$/°C. The novelty of this work lies in the adoption of two-step thermal expansion determination for 3C-SiC using both experimental and computational techniques.

## 1. Introduction

Silicon carbide is presently being investigated as a promising material for the third generation of semiconductor materials after the first and second-generation relative material, due to fact that SiC possesses excellent physical and electronic properties, which has attracted the attention of the researchers [1,2]. In fact, SiC has wide band gap, high thermal conductivity, high critical electric field, excellent mechanical strength, low thermal expansion, which allowed to design an innovative semiconductor device with respect to silicon ones, in terms of high breakdown voltage, restricted on-resistance and extreme operating temperature [3,4,5,6], such as space exploration, geothermal wells, and nuclear power instrumentation. Due to its electrical, mechanical, and thermal qualities, SiC is an excellent material for high-temperature pressure sensor devices [7,8,9]. It is well-known for its many polytypic forms that emerge under ambient conditions, SiC material has more than 250 poly-types, including 3C-SiC, 4H-SiC, 6H-SiC depending on the stacking sequence [10]. The zinc-blende (B3) polytype, also known as the 3C polytype, and the hexagonal wurtzite structured 6H polytype are the most widely studied and/or naturally occurring structures. The cubic structure is often referred to as beta (β) SiC, whilst the hexagonal and rhombohedral structures are also classified as alpha (α) SiC [11].

The coefficient of thermal expansion (CTE, α) of a material defines how its length changes in response to temperature. Matching the CTE of components enhances the robustness, dependability, and lifetime of devices in electrical and mechanical devices by reducing the chance of internal residual stresses forming due to temperature cycling [12,13,14,15,16]. In contrast, component mismatch CTE can jeopardies the device’s strength and integrity [17,18,19], causing temperature variations to render devices unusable, especially in electronics and high-precision applications [20,21,22]. As a result, strategies for designing materials with customized CTE are especially suitable for applications in electrical systems. Several works in [23,24,25,26,27] have investigated cubic (3C) or beta polytype SiC coefficient thermal expansion for approximation range extrapolation from ambient temperature to 1400 °C. Table 1 highlights prior research on the thermal expansion characteristics of SiC using X-ray diffraction [23,25,26,27] and dilatometry [24]. The thermal expansion coefficient values of 4.3–6.2 × 10^−6^ K^−1^ have been observed on average. For more details, refer to the work of [27], which is the most often cited in the study of the thermal expansion of 3C-SiC.

First-principles phonon calculation using quasi-harmonic approximation has found an excellent method to estimate thermal expansion at higher temperature for a several number of materials [28,29]. The lattice vibration of β-Si1-xC has studied at higher temperature without Gruneisen parameters [30]. Thermal expansion using phenomenological lattice dynamical theory in the quasi-harmonic approximation [31]. Another studied showed that, 3C-SiC at higher temperature and pressure up to 70 GPa predicted to phase transform to rock-salt phase (B1) due to the volume collapsed of around 18.1% [32].

This study aims to investigate coefficient thermal expansion based in situ X-ray crystallography at high temperature range from 25–800 °C at maximum peak (111) of 3C-SiC using both the experimental and computational approaches. We aim to compare the result obtained from our approach with the available data in the literature of coefficient thermal expansion of 3C-SiC. To the best of our knowledge, the study is the first to use both experimental at high temperature and the Gruneisen formalism theory along with density functional theory to calculate the thermal expansion of 3C-SiC.Morever, Mechanical and thermodynamics of 3C-SiC also investigated under at variance of pressure 0, 1 and 2 GPa. 

## 2. Materials and Methodology 

**Figure 1:**
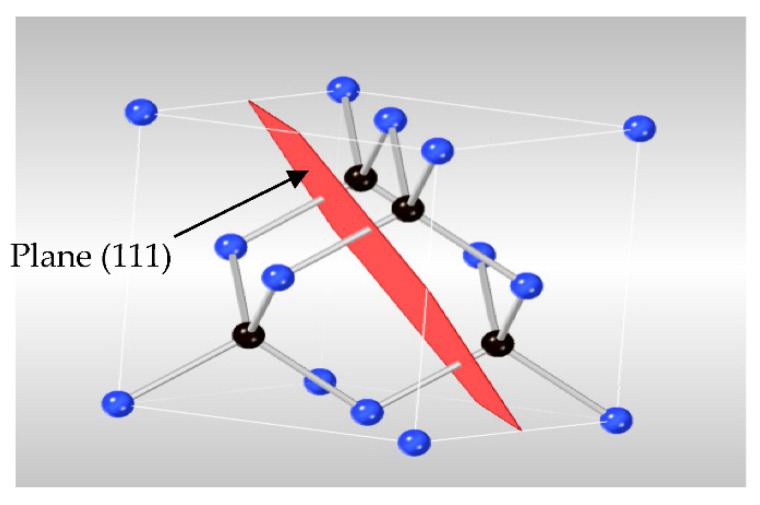
3C-SiC face-cantered cubic crystal. Blue and black ball are the silicon and carbon, respectively.

**Figure 2:**
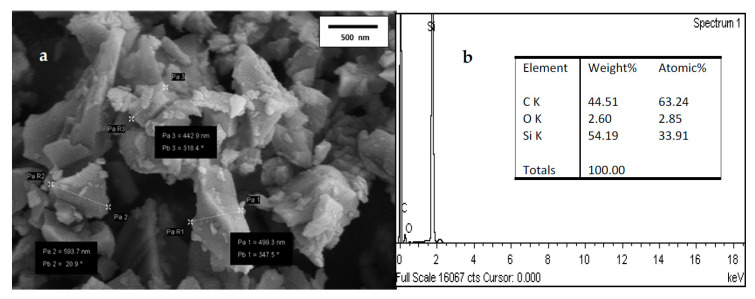
(**a**) FESEM of 3C-SiC nanoparticles. (**b**) EDX of 3C-SiC nanoparticles.

The 3C-SiC (99.9%) was purchased from Hong Wu International Group Ltd., Guangzhou, China. At room temperature, 3C-SiC has a face centered cubic structure (FCC). Its space group is F43m, as shown in Figure 1. Field Emission Scanning electron microscope (FESEM) used to characterize the powder of 3C-SiC. Image of FESEM test of 3C-SiC was conducted at Universiti Teknologi Petronas. The sample zoom magnification began with rang from ×1 k to ×100 K (High contrast mode) for optimized imaging and particle size of the sample morphology as illustrated in Figure 2. The determination of the chemical composition of 3C-SiC nano-powder was used Energy-Dispersive X-ray Spectroscopy (EDX) (SUPRA 55VP from Carl Zeiss AG Oberkochen, Germany). In-situ X-ray diffraction used to compute the expansion of maximum 2θ (θ = 20–80°) at peak (111) with varying the temperature. An X-ray CuKα incident beam with a wavelength of 0.154 nm was used for the XRD experiments. The device has been meticulously calibrated through the observation of standard (SRM660c) and by visual diffraction analysis of samples undergoing solid-sates structural transition. A Pt10Rh-Pt (10% rhodium) type S thermocouple (± 0.0025 °C tolerance) controlled oven chamber ensures a steady and homogeneous temperature distribution in the sample. The experiment was carried out in consecutive stages of 25 °C up to 800 °C, for a total of 9 steps. Before beginning the temperature dependent XRD measurement, the chamber was vacuumed under (10^−4^ mbar inert gas/argon).

### 2.1. Model 

The following equation was used to compute the face-centered cubic crystalline structure dimension of (a) of the unit cell, the volume of the cell, and the atomic radius:

(1) 2ksinθ=nλ

Here, *k* indicates to the d-spacing (m), *θ* for scattering angle (°), n represents the positive integer, and *λ* is the wavelength (m).

(2) $$\frac{1}{d_{hkl}^{2}}=\frac{h^{2}+k^{2}+l^{2}}{a^{2}}$$

where (*a*) is the lattice parameter of face-centered cubic crystalline structure dimension, and (k, h,  and l) are the Miller indices (m). There are 4 atoms per unit cell in a face closed centered cubic, and the relation to compute the atomic packing factor as given in [33] is:

(3) $$APF=\frac{\pi }{3\surd 2}$$

The atomic volume is obtained using:

(4) $$APF=\frac{\;\mathrm{volume}\;\mathrm{of}\;\mathrm{atomic}\;}{\;\mathrm{unit}\;\mathrm{cell}\;\mathrm{volume}\;}$$

Since the lattice parameters obtained from the Equation (2) has polynomial curve as function of the temperature, we fit the curve with third order polynomial to determine the lattice parameter as function of temperature that can be expressed as:

(5) $$a=-2.048\times 10^{-12}T^{3}+1.834\times 10^{-8}T^{2}+9.892^{-6}T+4.359$$

$$R^{2}=0.9965$$

The coefficient thermal expansion of 3C-SiC calculated using the derivatives of Equation (5) according and fitted to Equation (6) below, assuming one-dimensional of lattice parameter change with temperature and divided by lattice parameter.

(6) $$\alpha_{a}=\frac{d\left(Ina\right)}{dT}\approx \frac{1}{a_{0}}\times \frac{da}{dT}$$

where is a and $a_{0}$ is 3C-SiC lattice parameter values of the temperature and room temperature, respectively. 

The Scherrer’s equation used to estimate the crystallite size (*D*) is given as [34]:

(7) $$D=\frac{K\lambda }{\beta cos\theta }$$

where, k is the shape factor and its value is 0.9, λ is the wavelength, β  is full width at half maximum (FWHM), and θ is the diffraction angle. The strain induced (ε) in particles due to crystal imperfection and distortion can be calculated using the formula [34]:

(8) $$\varepsilon =\frac{\beta }{4\tan\;\left(\theta \right)}$$

Assuming that the particles size and strain to line boarding are not dependent to each other, the observed line breath is simply the sum of Equations (7) and (8).

(9) $$\beta =\frac{k\lambda }{D\;cos\theta }+4\varepsilon \;tan\theta$$

By rearranging Equation (9)

(10) $$\beta cos\theta =\frac{k\lambda }{D}+4\varepsilon \;sin\theta$$

The Equation (10), is Williamson-Hall equation and a plot is drawn with *4sinθ* along *x*-axis and *βcosθ* along y-axis for each temperatures of 3C-SiC. The data was linearly fitted, and the particle size estimated from the y-intercept and the strain indued value (ε) from the slop. 

### 2.2. Computational Details 

The phonon modes of 3C-SiC were calculated via first principles using the density function theory at frequency of 0 GPa, 1 GPa and 2 GPa. Materials Studio Version 6.0 (Accelrys) software was used in this research work [35]. Furthermore, DFT computations also incorporate structural optimization and enthalpy, including exchange–correlation functions using the Perdew–Burke–Ernzerho method [36]. Projection augmented plane wave (PAW) has been used to compute the interconnection between the actual electron and the valence electron of the ion [37]. Broyden, Fletcher, Goldfarb, and Shanno (BFGS) was utilized for the structural relaxation [38]. The energy cutoff of 500 eV was employed with plane wave basis set and Monkhorst pack technique of a 4 × 4 × 4 k point grid [39]. During the structural relaxation, the total energy of the model was reduced to a value less than 1.0 × 10^−6^ eV, the atom displacement to a value less than 0.001, the residual forces to a value less than 0.02 eV/Å, the residual bulk stress to less than 0.02 GPa. 

## 3. Results and Discussion 

### 3.1. In Situ X-ray Diffraction Result

**Figure 3:**
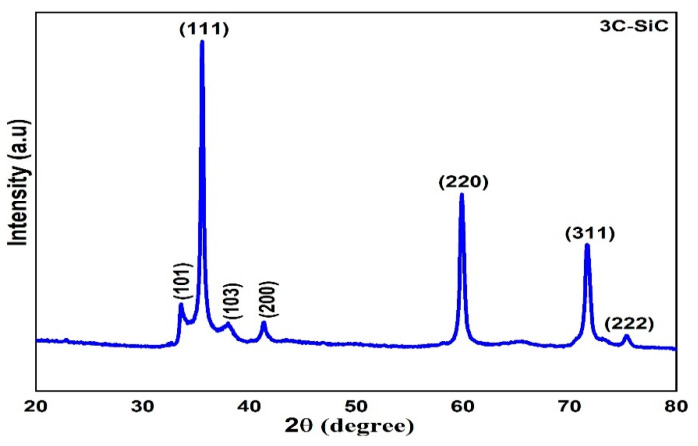
A representative powder XRD of 3C-SiC under ambient circumstances.

**Figure 4:**
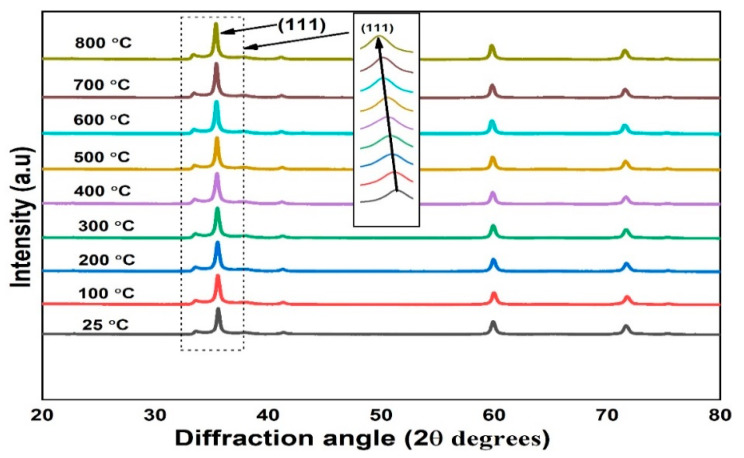
XRD patterns of 3C-SiC under different temperatures.

Figure 2 shows the image of the particle shapes of 3C-SiC powder at room temperature and the identifying the elementals composition of 3C-SiC. Figure 2a, the image confirms that the particles sizes are in nanoscale with agglomeration and Figure 2b, shows 3C-SiC has lowest impurities of O_2_ at weight of 2.6%. However, from EDX analysis is confirmed that highest proportion is carbon and silicon with percentage of 43.21% and 54.19%, respectively. fit2d software has been used to analyze the XRD diffraction pattern [40]. The highest temperature of the experiment is 800 °C. The diffraction patterns of 3C-SiC at room temperature and different temperature were represented in Figure 3 and Figure 4. Throughout the heating cycle, XRD patterns of 3C-SiC were obtained within the temperature range from 25 °C to 800 °C at 25 °C and from 100 °C at equal 100 °C steps. As seen in Figure 4, when the temperature increased, the patterns revealed a typical transition of maximum peaks (111) to lower 2*θ* angles, denotes to an increase of the inter-distance planner caused by thermal expansion. Table 2 presents the lattice parameters (a), 2*θ*, and volume.

**Figure 5:**
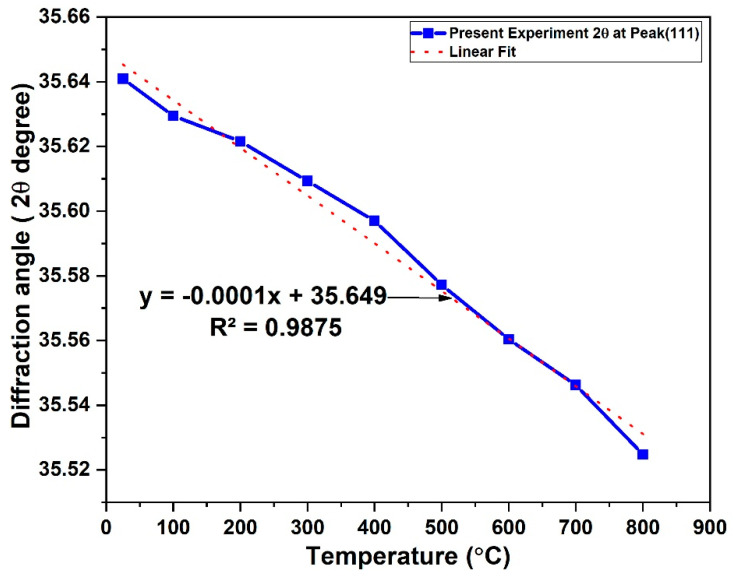
Peak broadening at peak (111) as function of temperature.

**Figure 6:**
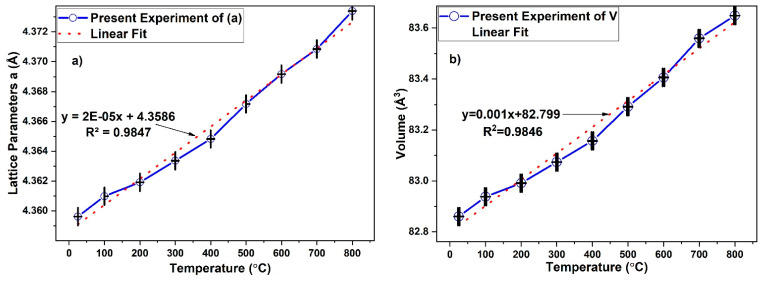
Crystalline structure of 3C-SiC: (**a**) lattice parameters and (**b**) unit cell volume.

At the 3C-SiC peak (111), the 2*θ* value decreases slightly with increasing temperature as presented in Figure 5. This is due to the thermal expansion that occurred in crystal lattice. The incident X-ray beam strikes a deeper depth of the crystalline thickness and the intensity in the Bragg positions diminishes. This is because of the thermal expansion diffuse scattering (i.e., electron-phonon). Lattice constant and cell volume were calculated based on the face-centered cubic closed packed structure of 3C-SiC model as presented in Table 2. Thermal expansion causes a nearly linear rise in the unit cell volume of beta silicon carbide over the whole temperature range investigated, as shown in Figure 6. The error bar indicated there is statistically significant change between the lattice parameter and volume as function of temperature. This trends which agree with previous works [23,24,25,26,27].

**Figure 7:**
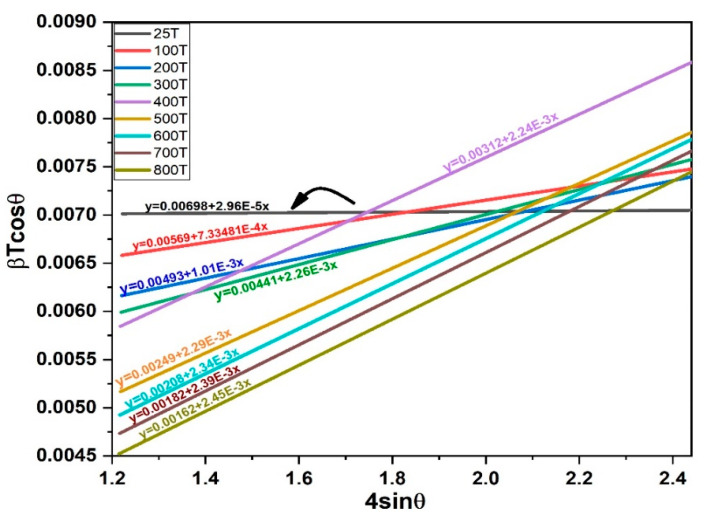
Williamson-Hall plot for 3C-SiC for different temperatures.

The scattered data of The Williamson-Hall (W-H) method are analysed in Figure 7. The (W-H) provided the macrostrain and inverse of the plot intercept estimate the particle size. The average particle size estimated from (W-H) at 25 °C is 0.00698 and increase as the temperature increased due to dilation of the crystal lattice as confirmed in Figure 6a,b. However, this trend is agreed with literature [41]. The lattice strain of 25 °C, 100 °C and 300 °C is fluctuation from $2.96\times 10^{-5}$, $7.33\times 10^{-4}$ and $1.01\times 10^{-3}$, respectively. However, from the temperature range of 400–800 °C, the lattice strain observed stable due to the crystalline structure stability.

### 3.2. Hybridization and Crystallization of 3C-SiC

**Figure 8:**
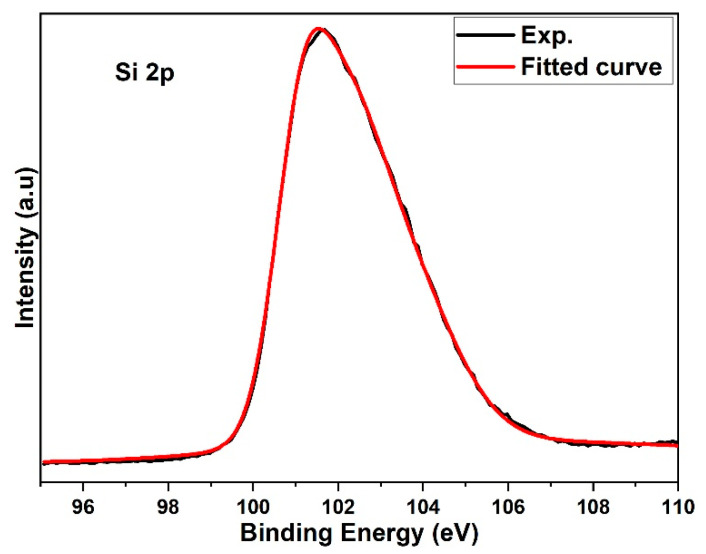
XPS spectrum of Si p2 of 3C-SiC nanoparticles from the surface of the crystalline and gaussian fitting represented by dashed line.

**Figure 9:**
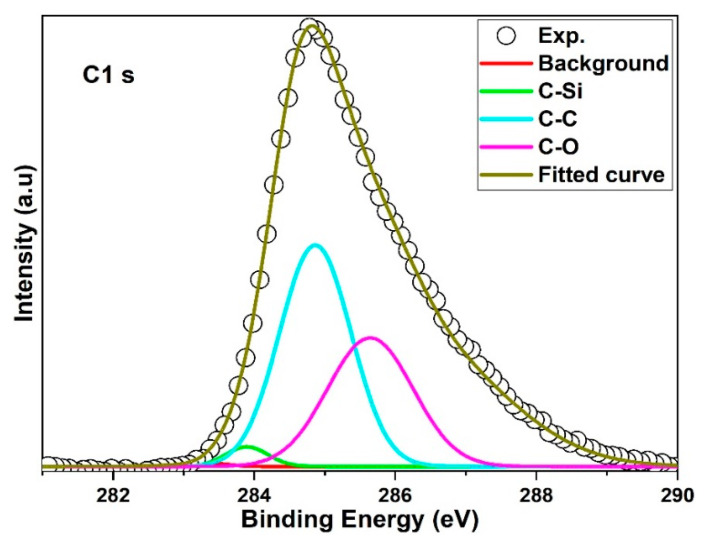
XPS of C 1s spectrum.

As it shown in Figure 8, XPS the spectrum contains of Si p2 which is denoted of by a solid line. Si p2 peaks has a high binding energy and asymmetric shape that indicative of the SiO_2_ found on 3C-SiC surface [42]. A peak of 101.5 eV refers to the binding energy of the Si-C bond corresponding to the recorded SiC crystal values in [43]. Gaussian dash line in the spectrum which on Si 2p spectrum is indicative to clean (not oxidized) of SiC surface which is representing of contribution of the 2p3/2-2p1/2 doublet components of Si bound to C in SiC lattice [44]. Figure 9 shows the C 1s spectrum recorded is slightly asymmetric to the peak because of different coordination patterns of carbon in 3C-SiC. The C 1s XPS peak can be Gaussian fitted into three peaks with the aid of Origin software. The initial peak at 283.3 eV in 3C-SiC represents C-Si bond in well crystallized SiC [45]. The carbon with activity in crystal lattice caused the formation of C-C peak bond at 283.9 eV and another peak at 285.6 eV, it is indicative of C-O bond in adsorptive CO_2_ impurities [46].

### 3.3. Experimental Technique of Thermal Expansion

**Figure 10:**
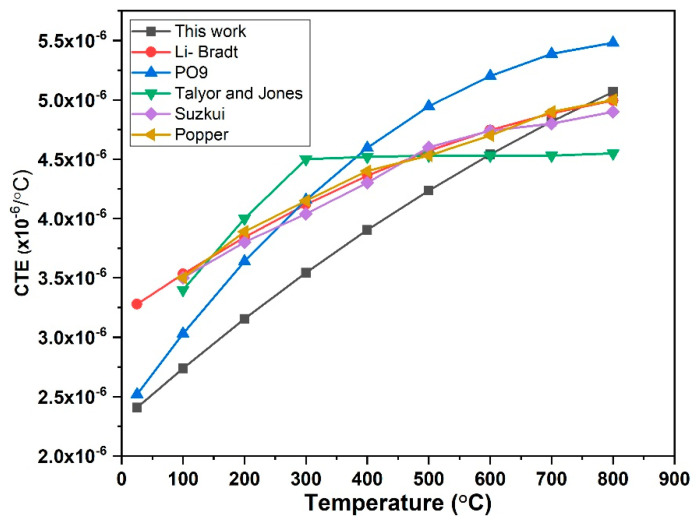
Illustrates the variation of the thermal expansion coefficient of 3C-SiC.

Temperature variation of the coefficient thermal expansion of 3C-SiC as computed in the present study and available data from the literature illustrated in Figure 10. It also compares the findings of this study with result of previous studies of [23,24,25,26,27]. The coefficient thermal expansion at room temperature was found 2.4 × 10^−6^/°C which nearly identical to room temperature of the value of 2.5 × 10^−6^/°C that reported by [26], and slightly closed to value of 2.9 × 10^−6^/°C of that reported by [24] and deviated to the values of 3.26 × 10^−6^/°C, 3.21 × 10^−6^/°C, 3.24 × 10^−6^/°C that reported by [23,25,27], respectively. The chemical structure of the molecule, the packing structure in the crystal lattice, and the chain arrangement all affects the coefficient of linear thermal expansion and its anisotropy [47]. The coefficient thermal expansion continuously increases to about 5.1 × 10^−6^/°C at 800 °C which is also compatible with the results of [24,25,27], expect for [26], which deviated increased to 5.48 × 10^−6^/°C and decreased of 4.5 × 10^−6^/°C in [23]. However, Ref. [23] have reported that the coefficient thermal expansion of 3C-SiC has a specific temperature dependence. Their findings indicate that the coefficient of thermal expansion of 3C-SiC rapidly increases below a certain threshold of 200 °C, but then remnant steady at about 4.5 × 10^−6^/°C over a wide temperature range from about 300–800 °C. The linear thermal expansion that has reported by [27], Refs. [24,25] mostly identical about 100 °C to 800 °C. However, this study reports a lower coefficient of the thermal expansion which has average 3.82×10^−6^/°C. The reason of this lower maybe due to technique for the synthesis of 3C-SiC because which [24,25,27] has not mentioned what technique that has been synthesize of 3C-SiC. For [26], the authors have investigated the thermal expansion under tri structural isotropic TRISO and as their XRD showed a different material not only3C-SiC, so maybe their result obtained measured with a high deviation of thermal expansion. Moreover, during the synthesize of beta silicon carbide at higher temperature, it has been observed a small amount of other polymorphism of silicon carbide such hexagonal (6H) [48]. However, hexagonal silicon carbide which exhibits anisotropic behavior in thermal expansion between the a-axis and c-axis [27,49] in where 3C-SiC exhibited an isotopically thermal expansion along a-axis and linear coefficient thermal expansion strictly followed a linear behavior. 

### 3.4. Computational Technique of Thermal Expansion

To measure the thermal expansion from the first principal calculation, the volume of thermal expansion must be calculated. The volume of thermal expansion ($\alpha_{v})=\frac{\gamma C_{v}}{3BV}$, where $C_{v}$ is a constant volume of specific heat, γ Gruneisan parameter, *B* is bulk modulus and Vm molar volume of 3C-SiC at specific temperature. The formula for calculating the sum of specific heat at constant volume can be $C_{v}=\frac{1}{4}\sum^{\;}p_{i}C_{i},\;$ where $C_{i}$ is the specific heat contribution from a single mode of frequency $\omega_{i}$ and $p_{i}$ is degeneracy, i.e., number of phonons or branches of frequency $\omega_{i}$ in the phonon dispersion curves. Since the 3C-SiC is face centered cubic which have four formula units that represent by factor $\frac{1}{4}$ in above equation. $C_{i}=R\left[x_{i}^{2}\exp\left(x_{i}\right)\right]/{\left[\exp\left(x_{i}\right)-1\right]}^{2}$, where $X_{i}=\hbar \omega_{i}/k_{B}T$, where $k_{B}$ is the Boltzmann constant and R is the gas constant. We have calculated the phonon frequencies of geometry optimized of 3C-SiC using CASTEP Module. The $C_{v}$ calculated of optic mode of 3C-SiC is 107 J/mol K.

To compute $\alpha_{V}$, first we need to evaluate $\gamma_{av}$ defined as $\gamma_{av}=\frac{\frac{1}{4}\sum^{\;}p_{i}c_{1}\gamma_{i}}{C_{v}}$. Gruneisen parameter mode is defined [50,51] as $\gamma_{i}=-\frac{\partial \ln\omega_{i}}{\partial \ln V}=\frac{B}{\omega_{i}}\frac{\partial \omega_{\gamma }}{\partial P}$, where $\omega_{i}$ is the frequency of the *i*th mode, B is the bulk modulus, P is the pressure, and V is the volume. Bulk modulus the of 3C-SiC is 212 Gpa which agree to previously calculated [52]. CASTEP code were used to calculate phonon frequencies at 0, 1, and 2 Gpa. $d\omega_{j}/dP$ has been used to calculate the mode Gruneisen parameters of all optic mode that obtained from the phonon frequencies. The calculated Gruneisen parameter average is 1.0483. The volume coefficient of thermal expansion is 0.33 of the lattice coefficients of thermal expansion ($\alpha_{L}$) [53] and computed using Gruneisen formalism approximately 2.2 × 10^−6^/°C which nearly closed to the experimental coefficient of thermal expansion that was calculated using a high temperature X-ray diffraction (reported in this study). Computation coefficient of thermal expansion of 3C-SiC were compared with other works in the literature as illustrated in Table 3. All computed values slightly closed to the computed value on this work. 

## 4. Mechanical and Thermodynamic Properties of 3C-SiC

As an important structural component employed in high-temperature applications such as the nuclear industry, the mechanical properties, and thermodynamics of 3C-SiC are very important. Table 4 shows the mechanical properties of 3C-SiC calculations, the result of our calculations is in good agreement with result of others theoretical calculation and experimental data. 

**Figure 11:**
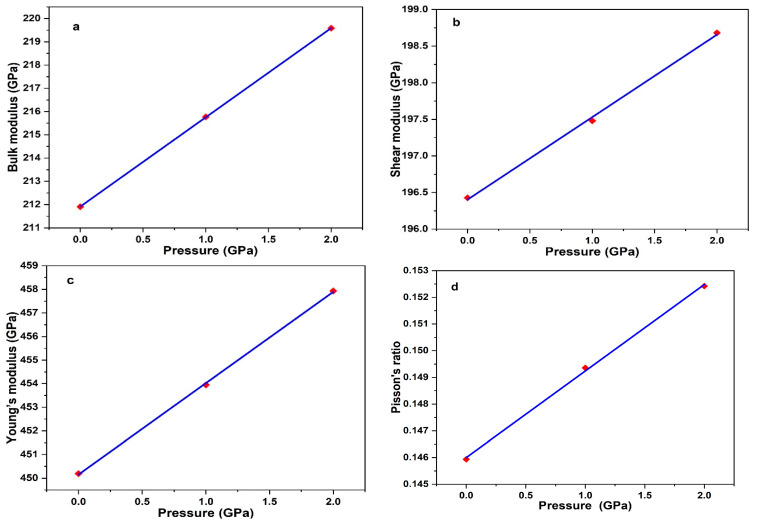
Mechanical properties of 3C-SiC based on different pressures; bulk modulus (**a**), shear modulus (**b**), Young’s modulus (**c**) and Poisson’s ration (**d**).

Figure 11 shows the mechanical properties of 3C-SiC with different pressure 0 Gpa, 1 Gpa and 2 Gpa. Overall, the values of the Bulk modulus, Shear modulus, Young’s modulus and Poisson’s ratio increase as the pressure increase. However, this trend has been found in [31] when 3C-SiC studied under high pressures. To our knowledge, the material is ductile if the value of G/B is smaller than 0.57, and the ductility of a material increases when the value of G/B decreases. The material is brittle if the value of G/B is larger than 0.57, the larger the value, the greater the brittleness of the material. 3C-SiC is brittle since that the value of G/B is greater than 0.57. Poisson’s ration is a parameter to characterize the brittleness and ductility of materials. When the Poisson’s ration > 0.33, the materials is ductile and when Poisson’s ration < 0.33 is brittle [57]. The computed Poisson’s ration shows that the brittleness of 3C-SiC under 0 Gpa, 1 Gpa and 2 Gpa as confirmed by the ratio of G/B. 

**Figure 12:**
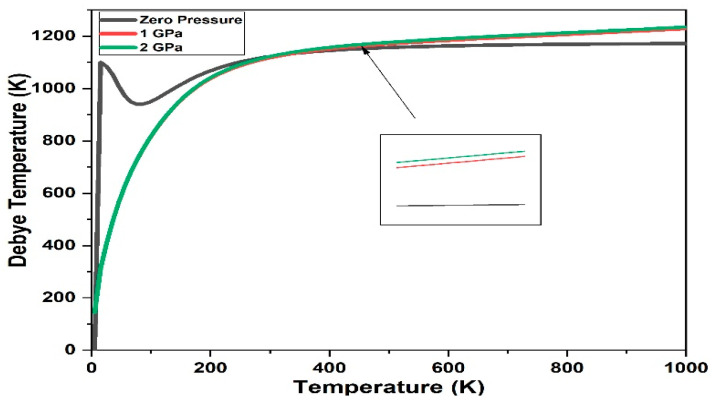
Debye temperature 3C-SiC.

Thermodynamic properties were carried out to investigate the variation of thermal properties with different pressures, such as Debye temperature, enthalpy, free energy, entropy, and heat capacity. Debye temperature represents highest mode of vibration of the crystal, during phonon vibrations [58]. Figure 12 shows the Debye temperatures of 3C-SiC with variation of the pressure 0 GPa, 1 GPa and 2 GPa. The result concludes that, the Debye temperature enhances with the enhancement of pressure and the slope of this increase as pressure increase. Similar behavior found in theoretical and experiment data [58]. 

**Figure 13:**
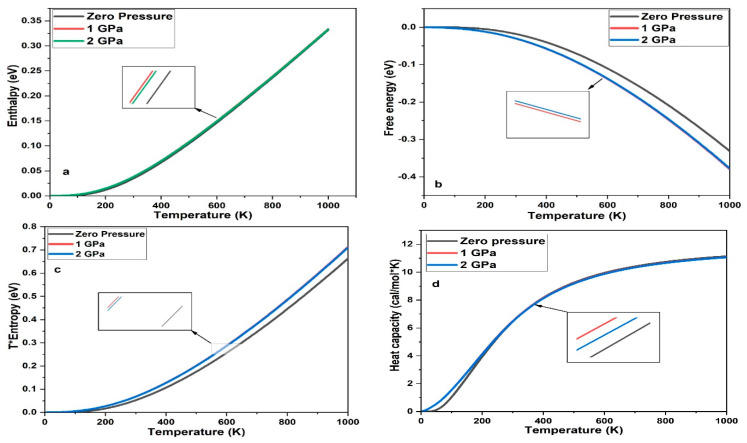
Enthalpy (**a**), free energy (**b**), entropy (**c**) and heat capacity (**d**) of 3C-SiC at different pressure.

Based on Debye quasi-harmonic approximation, we calculated the enthalpy, free energy, entropy, and heat capacity of 3C-SiC with different pressure 0 GPa, 1 GPa and 2 GPa. Figure 12 shows the enthalpy, free energy, entropy, and heat capacity of 3C-SiC, respectively. As the pressure and temperature increase, the free energy slowly increases below 200K and increase rapidly after 300K for 0 GPa, 1 GPa and 2 GPa, respectively. The value of the free energy decreases as the pressure increases which mean that reducing the internal energy converts less work outside which have different manner as the defect increased in 3C-SiC [30]. T*Entropy of 3C-SiC increase as the pressure increase due to increase the internal energy as shown in Figure 13c. The heat capacity of 3C-SiC < 1200 K, the heat capacity is related to temperature, because of the anharmonic approximation of the Debye model. The anharmonic effect on the heat capacity is repressed at a higher temperature and is near to the Dulong–Pettit limit, which applies to all solids at high temperatures [54]. In overall, as the pressure increased, enthalpy, entropy, and heat capacity will be increased. 

## 5. Conclusions

The coefficient thermal expansion of 3C-SiC was calculated utilising density function theory calculations of phonon frequencies as a function of pressure based on Gruneisen formalism and in-situ XRD powder diffraction measurement at the temperature range of 25–800 °C. The coefficient thermal expansion was established to follow a second order polynomial relationship as temperature increase $\;(\alpha_{11}=-1.423\times 10^{-12}T^{2}+4.973\times 10^{-9}T+2.269\times 10^{-6}$). At 25 °C, the coefficient thermal expansion is found 2.4 $\times \;10^{-6}$/°C which is identical with computed from the first principal calculations of phonon frequencies that closed to 2.2 $\times \;10^{-6}$/°C. However, the quasi-harmonic approximation based on the density function theory is still a valid methodology for predicting the physical properties of material.

## Figures and Tables

**Table 1:** Average coefficients of thermal expansion of 3C–SiC (adapted from [26]).

Author	Technique	Temperature Range (°C)	α (10^−6^/K^°^)
Li, Bradt [27]	XRD	RT-1000	4.45
Taylor, Jones [23]	XRD	RT-1200	4.4
Popper, Mohyuddin [24]	Dilatometer	RT-1400	4.4
Suzuki et al. [25]	XRD	RT-800	4.3
Ngoepe, de Villiers [26]	XRD	RT-1200	-

**Table 2:** The lattice parameters and volume, of 3C-SiC as variant of temperature.

Temperature (°C)	*a* (Å)	2*θ*°	Volume (Å)^3^
25	4.35962	35.64	82.86
100	4.36098	35.63	82.94
200	4.36191	35.62	82.99
300	4.36336	35.61	83.07
400	4.36482	35.60	83.15
500	4.36717	35.58	83.29
600	4.36917	35.56	83.41
700	4.37085	35.55	83.56
800	4.37341	35.53	83.65

**Table 3:** Experimentally and computationally coefficients of thermal expansion of 3C-SiC.

Symbol	$\alpha_{l}$ Experimental RT (×10^−6^/°C)	$\alpha_{v}$ Computational RT (×10^−6^/°C)
3C-SiC (This work)	2.4	2.2
3C-SiC [52]	-	2.47
3C-SiC [31]	-	2.5

**Table 4:** The elastic constants (GPa), Bulk modulus B (GPa), Young’s modulus E (GPa) and shear modulus G (GPa).

3C-SiC	C_11_	C_12_	C_44_	B	E	G
Our Cal	383.4	126.1	241.6	212	450.19	196.42
Other Cal. [54]	383.3	125.2	239.6	211.3	432.9	186.9
Other Cal. [55]	384.5	121.5	243.3	209	437.6	190.1
Exp. [56]	380	142	256	225	448	192

## Data Availability

We are continually working on this project, and the data will be used for future research and analysis. However, any researcher who needs the data for further investigations can contact the corresponding author through email with reasonable justification.
